# Clinical presentation and treatment outcomes in a South African thrombotic thrombocytopenic purpura cohort with and without HIV

**DOI:** 10.4102/sajhivmed.v27i1.1795

**Published:** 2026-04-30

**Authors:** Nokubonga Vundla, Jenique Bailly, Karryn Brown, Jenna Oosthuizen, Estelle Verburgh

**Affiliations:** 1 Department of Medicine, Division of Clinical Haematology, Faculty of Health Sciences, University of Cape Town, Cape Town, South Africa; 2 Groote Schuur Hospital, Cape Town, South Africa; 3 Department of Pathology, Division of Haematology, Faculty of Health Sciences, University of Cape Town, Cape Town, South Africa; 4 National Health Laboratory Service, Cape Town, South Africa

**Keywords:** thrombotic thrombocytopenic purpura, haemolytic anaemia, thrombotic microangiopathy, HIV-associated TTP, ADAMTS13, treatment outcomes, plasma infusion, plasma exchange

## Abstract

**Background:**

HIV is the most common cause of secondary thrombotic thrombocytopenic purpura (TTP) in South Africa.

**Objectives:**

To assess the clinical presentations and outcomes of patients treated for HIV-associated and idiopathic TTP.

**Method:**

We conducted a retrospective cohort study of patients consecutively diagnosed with TTP from 2010 to 2020 at Groote Schuur Hospital. Patients were identified by reviewing hospital and Western Cape Blood Services records. Kaplan-Meier curves and log-rank tests were used to evaluate remission rates, both overall and by HIV status and treatment group. Logistic regression models were used to identify predictors of remission and relapse.

**Results:**

One hundred and thirty-nine patients were included, 85.6% of whom were HIV positive. There were no significant differences in the TTP pentad features by HIV status. Most patients achieved remission (71.9%) with a median time of eight days. Remission occurred significantly earlier in those treated with fresh frozen plasma only, suggesting less severe disease (median = 8 days [interquartile range 6–10]), compared to those requiring plasma exchange, suggesting more severe disease (median = 12 days [interquartile range 8–22]). The overall mortality in the 10-year period was 38.9%, with 10.8% of the surviving patients relapsing after 6 months. There were no significant differences in remission status, time to remission, mortality or relapse by HIV status. All HIV-positive patients who relapsed had defaulted their antiretroviral therapy (ART).

**Conclusion:**

HIV status did not affect patient outcomes in our cohort. ART is important in preventing HIV-associated TTP and relapse.

**What this study adds:** A well-defined demographic of patients with TTP in the ART era in South Africa.

## Introduction

Thrombotic thrombocytopenic purpura (TTP) is a rare, life-threatening thrombotic microangiopathy (TMA) characterised by widespread deposition of platelet-rich microvascular thrombi, consumptive thrombocytopenia, and microangiopathic haemolytic anaemia (MAHA), leading to target organ ischaemia and dysfunction.^1,2,3,4^ Severe ADAMTS13 (a disintegrin and metalloprotease with thrombo-spondin type 1 motif, member 13) deficiency is considered a hallmark of TTP and results in accumulation of ultra-large Von Willebrand factor multimers, driving platelet aggregation and microvascular thrombosis.^5,6^

TTP is uncommon, with an estimated annual incidence of one case per million worldwide.^6^ Congenital TTP caused by genetic mutations in the ADAMTS13 gene accounts for approximately 5% of cases whereas the majority of cases are acquired, most often mediated by autoantibodies against ADAMTS13.^7^ Secondary causes of TTP include HIV infection, autoimmune diseases, transplantation, and pregnancy.^8^ In a study by Moola et al., the incidence of HIV-associated TTP in South Africa was estimated at 17.6 to 63.8 cases per million population, based on South African National Blood Service prescribing patterns for plasma exchange (PEX).^9^

Early diagnosis of TTP relies on laboratory evidence of TMA. Most patients present with a triad of severe thrombocytopenia, marked fragmentation haemolysis, and elevated lactate dehydrogenase (LDH), which is often sufficient for a presumptive diagnosis.^6,10^ Definitive diagnosis requires severely reduced ADAMTS13 activity (< 10% of normal).^11^ In HIV-associated TTP, however, normal ADAMTS13 activity does not exclude TTP.^3^ Additionally, ADAMTS13 testing is typically unavailable during the acute phase of TTP treatment, as it is performed only at specialised reference laboratories in South Africa and often batch tested.^12^

HIV infection significantly increases the risk of TTP. In South Africa, most cases are HIV-associated, frequently representing the initial presentation of HIV.^11,13,14^ Unlike idiopathic TTP, HIV-associated cases may occur even with normal ADAMTS13 activity, and the underlying pathogenesis remains incompletely understood.^5,15,16^ Although advanced HIV (low CD4+ T-cell count and AIDS) is a recognised risk factor for TTP, a decline in HIV-associated TTP during the era of antiretroviral therapy (ART) has not been demonstrated in South Africa.^15^ Since the induction of ART in the early phase of HIV infection in 2015 under the ‘Test and Treat’ strategy,^17^ it remains unclear whether the annual incidence of HIV-associated TTP has decreased.

Clinical presentation in TTP is variable, with the classic pentad of thrombocytopenia, MAHA, renal impairment, neurological dysfunction, and fever observed in only a minority of patients.^15,18^ In the context of HIV, baseline organ dysfunction may further complicate the clinical picture.^15,18^

The current standard of care for TTP in South Africa includes daily therapeutic PEX with plasma replacement, or daily large volume (30 mL/kg/day) plasma infusion (PI), often combined with oral corticosteroids. These treatment modalities have substantially reduced mortality of patients with TTP.^19^ However, treatment protocols vary, and outcomes in HIV-associated TTP in the era of widespread ART in South Africa have not been fully characterised. Some centres in South Africa have reported their experiences with managing TTP in retrospective studies and these studies looked at cohorts treated prior to, or very early during, the ‘Test and Treat’ era of HIV management.^13,19,20^

In this study, we aimed to assess the clinical presentations and outcomes of patients treated for HIV-associated and idiopathic TTP at a tertiary healthcare facility in South Africa, providing insight into disease patterns and treatment effectiveness in the ART era.

## Research methods and design

This was a single-centre, retrospective cohort study conducted at the Clinical Haematology Unit at Groote Schuur Hospital, a tertiary healthcare facility in Cape Town, South Africa. All patients diagnosed with idiopathic and HIV-associated TTP over a 10-year period between 01 January 2010 and 31 December 2020 were included in the study. Those patients with an identifiable cause of secondary TTP, for example pregnancy and autoimmune diseases, were excluded from the study.

The study participants were identified by reviewing inpatient and outpatient files as well as a list compiled by the Western Cape Blood Services of all patients who received fresh frozen plasma (FFP) during the study period at Groote Schuur Hospital with the keywords TTP, MAHA and TMA provided by the clinician on the request form. This list was correlated with patient medical records to identify patients with TTP. The diagnosis of TTP was based on laboratory investigations and required the presence of thrombocytopenia and fragmentation haemolysis in the absence of any other identifiable causes of TMA such as disseminated intravascular coagulation. Idiopathic TTP cases were identified by reviewing the clinical notes and laboratory results. Idiopathic TTP was defined as TTP in the absence of identifiable causes for TTP, such as HIV infection.

### Patient data

Data were collected from patient records and the local laboratory information system. Demographic and clinical data included patient age, sex, HIV status, ART status, neurological dysfunction, and whether fever was present at diagnosis, as well as the treatment provided. Management outcomes recorded included the time to remission, duration of hospital stay, relapse, and mortality. Mortality was further classified according to the probable cause and whether death occurred during the index hospital admission, post discharge, or during disease relapse.

### Treatment protocol at Groote Schuur Hospital

Patients were treated with supportive measures, daily large-volume FFP (30 mL/kg) administered by means of a central or peripheral line, and corticosteroid therapy. Diuretics were administered as clinically indicated to manage or prevent fluid overload. FFP was given daily until clinical improvement in neurological function and laboratory endpoints were achieved (platelet count > 150 × 10^9^/L and LDH level < 450 U/L on two consecutive days). Thereafter, FFP was tapered by 320 mL (1 unit) every 3 days. As adjunctive therapy, ART-naïve HIV-positive patients were commenced on ART as per South Africa’s national ART guidelines.^20^

Patients refractory to the above regimen, and those unable to tolerate large-volume PIs because of fluid overload, were offered PEX with FFPs.^13,20^ On days when PEX was unavailable, mainly on weekends, FFPs were administered at 30 mL/kg/day.^20^ Haemodialysis was provided as indicated for acute renal failure.

### Laboratory investigations

Laboratory investigations performed at presentation and included in this study comprised full blood count, peripheral blood smear to identify and enumerate red cell fragments, LDH levels, renal function tests (urea and creatinine levels), HIV serology and, if HIV positive, the absolute CD4+ T-cell count and HIV viral load. ADAMTS13 activity and antibody tests were requested prior to FFPs in very few patients, and these results were not included in the analysis. Significant red cell fragmentation was defined as a red cell fragment count > 1%, significant thrombocytopenia as a platelet count < 90 × 10^9^/L, and an elevated LDH as > 190 IU/L. Creatinine > 100 µmol/L was considered a marker of renal dysfunction.^13,20,21^ Advanced HIV was defined by a CD4+ T-cell count < 200 cells/µL or stage IV AIDS-defining condition as per WHO guidelines.^22^

### Clinical outcome

Duration of hospital stay was noted, and complications such as nosocomial infections were documented. Remission was defined as a platelet count > 150 × 10^9^/L and LDH < 450 U/L on at least 2 consecutive days. Relapse was defined as recurrence of TTP after FFP treatment was concluded. Refractory TTP was defined as failure of improvement in laboratory parameters, namely platelet count, anaemia, and LDH.^20,21^ The causes of death were documented based on treating clinicians’ reports.

### Data capturing and analysis

All data were entered into the Clinical Haematology REDCap database. Data were exported from REDCap^®^ into STATA® Version 14 (Stata Corporation, College Station, Texas, United States) for analysis. Patient characteristics were described by frequencies (%), or medians and interquartile ranges. Categorical data were compared using Chi-squared and Fisher’s exact tests. The Mann-Whitney *U* test was used to compare continuous variables as they were not normally distributed. Kaplan-Meier curves were also used to display remission rates for the total cohort, by HIV status and by treatment group. Remission rates between groups were compared using the log-rank test. Univariable and multivariable logistic regression models were used to identify predictors of remission, time to remission and relapse. Statistical significance was set at *P* < 0.05 for all analyses.

### Ethical considerations

The study was approved by the University of Cape Town Human Research Ethics Committee (HREC approval number 112/2021) and institutional approval was obtained from Groote Schuur Hospital. A waiver of consent for data collection is in place for patients included in the Clinical Haematology REDCap database prior to 2019, and informed consent is obtained for patients included from 2019 onwards. The REDCap database is a secure password protected and access-controlled database. Patient personal identifiers were removed before data analysis to further ensure patient confidentiality. The study was conducted in accordance with the 2013 Helsinki Declaration.

## Results

**FIGURE 1:**
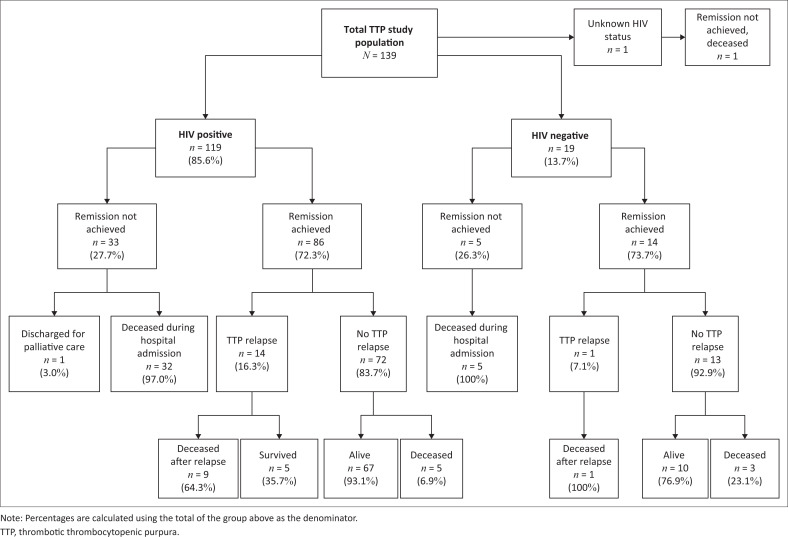
Flow chart of study patients and their outcomes.

Data from 139 consecutive patients admitted with HIV-associated or idiopathic TTP during the study period were reviewed. A flow chart of the study participants and their outcomes is presented in Figure 1. The baseline characteristics, treatment and outcomes are presented in Table 1 and Table 2. Most patients had HIV-associated TTP (85.6%), and the median age of the cohort was 34.0 years with most patients being women (73.4%). Of the HIV-positive patients, only one patient (0.8%) was on ART and virally suppressed, 49.6% (*n* = 59) were ART-naïve, and 49.6% (*n* = 59) had defaulted ART at the time of presentation. The median CD4+ T-cell count for the HIV-positive cohort was 154.5 cells/µL. There were no significant differences in age or sex between the HIV-positive and HIV-negative (idiopathic TTP) subgroups. The annual number of TTP cases, stratified by HIV status, is shown in Online Appendix 1 Figure 1-A1. There was no observed decline in the number of TTP cases over the study period.

**TABLE 1:** Baseline characteristics of patients diagnosed with thrombotic thrombocytopenic purpura between 2010 and 2020.

Characteristic	Total (*N* = 139)				HIV-associated TTP (*n* = 119)				HIV-negative TTP (*n* = 19)				*P*-value
	Median	IQR	*n*	%	Median	IQR	*n*	%	Median	IQR	*n*	%	
**Demographic data**													
Age (years)	34.0	28.6–40.0	-	-	33.6	28.3–38.8	-	-	41.2	32.6–49.0	-	-	0.014
Sex	-	-	-	-	-	-	-	-	-	-	-	-	-
Male	-	-	37	26.6	-	-	30	25.2	-	-	7	36.8	0.288
Female	-	-	102	73.4	-	-	89	74.8	-	-	12	63.2	-
**Features of classic TTP pentad**													
Fever	-	-	102	73.4	-	-	90	75.6	-	-	12	63.2	0.250
Thrombocytopenia	-	-	138	99.3	-	-	118	99.2	-	-	19	100.0	0.688
Haemolytic anaemia	-	-	136	97.8	-	-	116	97.5	-	-	19	100.0	0.484
Renal dysfunction	-	-	62	44.6	-	-	53	44.5	-	-	8	42.1	0.843
Neurologic dysfunction	-	-	123	88.5	-	-	107	89.9	-	-	15	79.0	0.166
All five features	-	-	30	21.6	-	-	29	24.4	-	-	1	5.3	0.061
**Laboratory data**													
Creatinine (μmol/L) (*n* = 135)	97.0	73.0–154.0	-	-	98.0	73.0–161.5	-	-	89.5	72.0–139.0	-	-	0.777
Haemoglobin (g/dL)	5.7	4.8–6.9	-	-	5.7	4.7–6.9	-	-	6.1	5.0–8.6	-	-	0.171
Platelets (x 10^9^/L)	14.0	8.0–24.0	-	-	14.0	8.0–24.0	-	-	14.0	9.0–24.0	-	-	0.894
LDH (IU/L) (*n* = 93)	1453.0	985.0–2121.0	-	-	1385.0	943.0–2121.0	-	-	1602.5	1254.0–2255.0	-	-	0.269
White cell count (x 10^9^/L)	9.2	6.5–13.1	-	-	8.9	6.4–13.0	-	-	9.7	8.2–14.4	-	-	0.292
MCV (fL)	89.0	83.0–97.0	-	-	89.0	83.0–98.0	-	-	88.0	81.0–93.0	-	-	0.453
CD4 count (cells/mm^3^) (*n* = 116)	-	-	-	-	154.5	86.0–242.0	-	-	-	-	-	-	-
**ART status**													
ART naïve	-	-	-	-	-	-	59	49.6	-	-	-	-	-
Defaulted ART	-	-	-	-	-	-	59	49.6	-	-	-	-	-
On ART, virally suppressed	-	-	-	-	-	-	1	0.8	-	-	-	-	-

Note: Values expressed as either median and IQR, or *n* and %. Only one patient had an unknown HIV status.

TTP, thrombotic thrombocytopenic purpura; LDH, lactate dehydrogenase; ART, antiretroviral therapy.

**TABLE 2:** Treatment and outcomes of thrombotic thrombocytopenic purpura patients by HIV status.

Characteristic	Total (*N* = 139)				HIV-associated TTP (*n* = 119)				HIV-negative TTP (*n* = 19)				*P*-value
	Median	IQR	*n*	%	Median	IQR	*n*	%	Median	IQR	*n*	%	
**Treatment**													
FFP only	-	-	113	81.3	-	-	98	83.0	-	-	14	73.7	0.327
FFP and PEX	-	-	25	18.0	-	-	20	17.0	-	-	5	26.3	-
Deceased before treatment	-	-	1	0.7	-	-	1	0.8	-	-	0	0.0	-
**Remission**													
Achieving remission	-	-	100	71.9	-	-	86	72.3	-	-	14	73.7	0.898
Achieving remission with FFP only	-	-	81	72.3	-	-	72	73.5	-	-	9	64.3	0.472
Achieving remission with FFP and PEX	-	-	19	76.0	-	-	14	70.0	-	-	5	100.0	0.160
**Time to remission (days)**													
Overall	8.0	6.00–12.00	-	-	8.00	6.00–12.00	-	-	8.00	5.00–11.00	-	-	0.708
FFP only	8.0	6.00–10.00	-	-	8.00	6.00–10.00	-	-	7.00	5.00–10.00	-	-	0.423
FFP and PEX	12.0	8.00–22.00	-	-	13.00	10.00–23.00	-	-	11.00	7.00–14.00	-	-	0.377
**Laboratory data at remission**													
Creatinine (μmol/L) (*n* = 80)	70.50	58.00–87.50	-	-	70.50	55.50–87.50	-	-	71.00	66.50–107.00	-	-	0.325
Haemoglobin (g/dL)	9.70	8.50–10.50	-	-	9.70	8.40–10.60	-	-	10.00	8.70–10.30	-	-	0.945
Platelets (x 10^9^/L)	283.00	227.50–363.50	-	-	286.00	231.00–368.00	-	-	259.00	200.00–300.00	-	-	0.338
LDH (IU/L) (*n* = 32)	308.50	263.00–344.50	-	-	308.50	260.00–344.50	-	-	316.00	283.50–410.00	-	-	0.200
Haematocrit (*n* = 45)	0.29	0.25–0.33	-	-	0.29	0.25–0.33	-	-	0.30	0.26–0.32	-	-	0.742
White cell count (*n* = 99)	7.20	5.3–9.30	-	-	7.00	5.00–9.10	-	-	7.40	6.70–10.10	-	-	0.144
MCV (*n* = 97)	94.00	90.00–101.00	-	-	95.00	90.00–102.00	-	-	92.00	89.00–100.00	-	-	0.435
**Outcome after initial episode**													
Survived	-	-	94	67.6	-	-	83	69.8	-	-	11	57.9	0.303
Deceased	-	-	45	32.4	-	-	36	30.3	-	-	8	42.1	-
**Length of stay (days)**													
Survived	20.00	15.00–27.00	-	-	20.00	14.00–27.00	-	-	24.00	17.00–41.00	-	-	0.352
Deceased during admission	5.00	2.00–11.00	-	-	5.00	2.00–11.00	-	-	3.00	2.00–16.00	-	-	0.561
**Number who relapsed**	-	-	15	10.8	-	-	14	11.8	-	-	1	5.3	0.398
**Time to relapse (days)**	169.00	146.00–281.00	-	-	186.00	149.00–281.00	-	-	87.00	87.00–87.00	-	-	0.247
**Final outcome**													
Deceased	-	-	54	38.9	-	-	45	37.8	-	-	8	42.1	0.496
Discharged from haematology	-	-	76	54.7	-	-	67	56.3	-	-	9	47.4	-
Lost to follow up	-	-	1	0.7	-	-	1	0.8	-	-	0	0.0	-
Presumed deceased	-	-	3	2.2	-	-	2	1.7	-	-	1	5.3	-
Relocated	-	-	5	3.6	-	-	4	3.4	-	-	1	5.3	-
**Cause of death**													
Severe TTP	-	-	44	81.5	-	-	36	80.0	-	-	7	87.5	0.341
Sepsis	-	-	7	13.0	-	-	7	15.6	-	-	0	0.0	-
Other	-	-	2	3.7	-	-	1	2.2	-	-	1	12.5	-
Unknown	-	-	1	1.9	-	-	1	2.2	-	-	0	0.0	-

Note: One patient deceased before treatment was administered. Only one patient had an unknown HIV status.

FFP, fresh frozen plasma; PEX, plasma exchange; LDH, lactate dehydrogenase; TTP, thrombotic thrombocytopenic purpura; MCV, mean corpuscular volume.

### Features of classic thrombotic thrombocytopenic purpura pentad

Of the classic TTP pentad (thrombocytopenia, haemolytic anaemia, renal dysfunction, neurologic dysfunction, and fever), renal dysfunction (creatinine above 100 µmol/L) was least common, occurring in < 50% (*n* = 62) of the study population (Table 1). Almost all patients presented with thrombocytopenia (*n* = 138, 99.3%) and haemolytic anaemia (*n* = 136, 97.8%). Neurologic dysfunction (*n* = 123, 88.5%) and fever with temperature exceeding 37.4 °C (*n* = 102, 73.4%) were also common. There were no significant differences between the HIV-positive and HIV-negative subgroups. However, more patients with HIV-associated TTP presented with neurological dysfunction (*n* = 107, 89.9% vs *n* = 15, 79.0%), showing a trend towards statistical significance (*P* = 0.166). Patients who did not achieve remission were significantly more likely to present with neurological dysfunction compared to those who achieved remission (*n* = 84, 84.0% vs *n* = 39, 100%, *P* = 0.008) (Online Appendix 1 Table 1-A1). No further significant differences between the features of the classic TTP pentad and remission status were observed.

### Laboratory data

Key baseline laboratory results are presented in Table 1. No significant differences were observed between the HIV-positive and HIV-negative subgroups. ADAMTS13 activity results were available for only six patients (4.3%), and meaningful statistical analyses could therefore not be performed using this variable.

### Treatment and outcomes

Most patients (*n* = 113, 81.3%) were treated with therapeutic FFP as per local protocol (Table 2). Only 25 patients (18%) required the addition of PEX, as their laboratory parameters did not improve. Regardless of the treatment protocol used (FFP only vs FFP with PEX), most patients (*n* = 100, 71.9%) achieved remission. No significant differences in treatment protocol and remission status were noted between the HIV-positive and HIV-negative subgroups. Univariable and multivariable logistic regression showed no statistical relationship between patient factors assessed (age, sex, and HIV status) or treatment protocol and remission status (Table 3).

**TABLE 3:** Univariable and multivariable logistic regression models for the outcome variable; remission.

Variable	Odds ratio	*P*-value	95% CI
**Univariable analysis**			
Age	1.01	0.768	0.97–1.04
Sex (female)	1.12	0.792	0.49–2.56
HIV status (positive)	0.93	0.898	0.31–2.79
Treatment (FFP and PEX)	1.25	0.662	0.46–3.42
**Multivariable analysis**			
Age	1.00	0.867	0.96–1.04
Sex (female)	1.23	0.634	0.53–2.86
HIV status (positive)	0.97	0.963	0.31–3.02
Treatment (FFP and PEX)	1.21	0.716	0.44–3.36

FFP, fresh frozen plasma; PEX, plasma exchange; CI, Confidence Interval.

**FIGURE 2:**
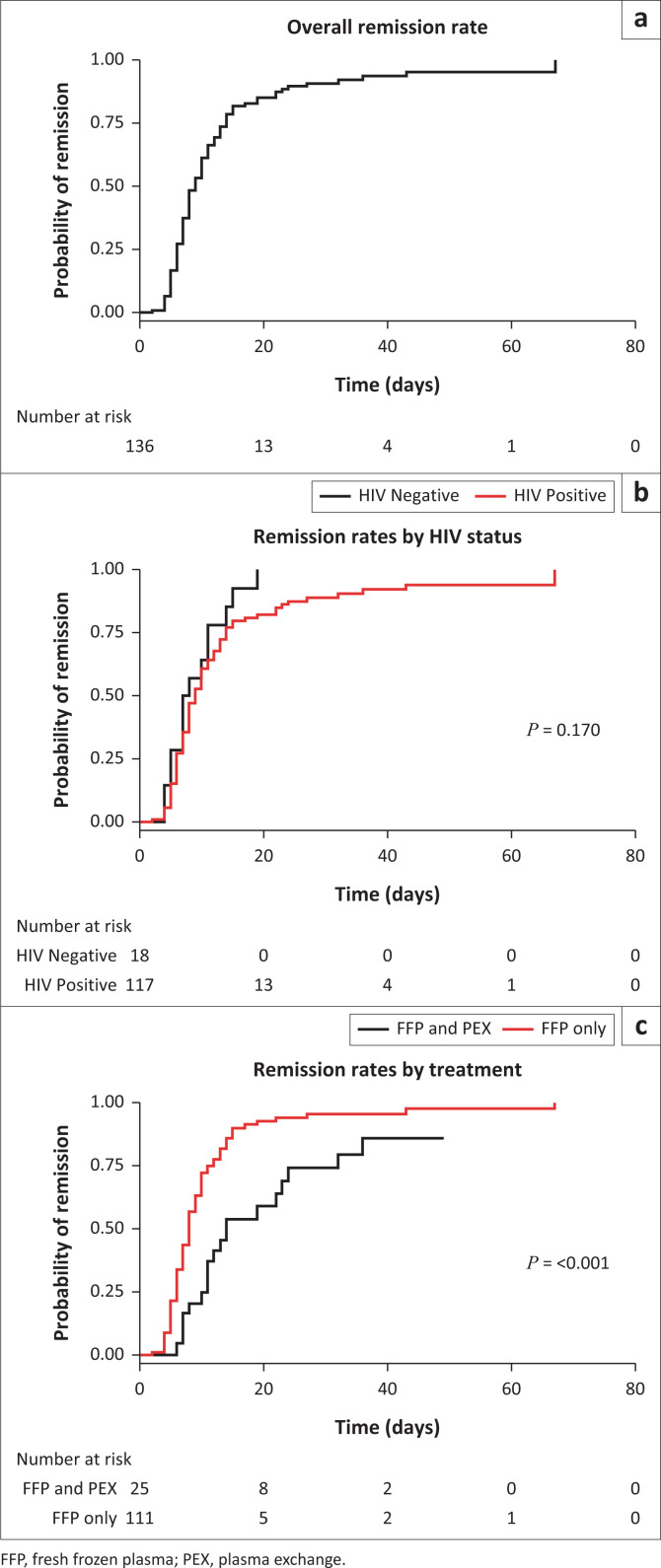
Remission rate of (a) the total TTP cohort, (b) by HIV status, and (c) by type of treatment received.

The overall median time to remission was 8 days (IQR 6–12) (Table 2 and Figure 2a). There was no significant difference in time to remission by HIV status (Table 2 and Figure 2b). Remission occurred significantly earlier in those treated with FFPs only (median = 8 days [IQR 6–10]) compared to those requiring the addition of PEX (median = 12 days [IQR 8–22]) (Table 2 and Figure 2c).

Univariable and multivariable linear regression models identified treatment protocol as a predictor of time to remission (Table 4). Patients treated with FFP plus PEX took an average of 5.9 days longer to achieve remission than those treated with FFP only, holding all other variables constant (*P* = 0.012). The laboratory results for all patients who achieved remission, regardless of HIV status, showed normal renal function and platelet counts at time of discharge (Table 2).

**TABLE 4:** Univariable and multivariable linear regression models for the outcome variable; time to remission (*N* = 100).

Variable	Co-efficient	*P*-value	95% CI
**Univariable analysis**			
Age	0.02	0.788	−0.15–0.19
Sex (female)	−0.37	0.857	−4.40–3.66
HIV status (positive)	1.88	0.464	−3.20–6.96
Treatment (FFP and PEX)	5.45	0.015	1.08–9.82
**Multivariable analysis**			
Age	0.01	0.928	−0.17–0.18
Sex (female)	−0.41	0.841	−4.42–3.60
HIV status (positive)	3.17	0.235	−2.10–8.43
Treatment (FFP and PEX)	5.86	0.012	1.34–10.39

FFP, fresh frozen plasma; PEX, plasma exchange; CI, Confidence Interval.

Following the first TTP episode, there were 54 deaths (38.9%), 45 (37.8%) among the HIV-positive subgroup, eight (42.1%) among the HIV-negative subgroup and one patient with an unknown HIV status. The median length of hospital stay for survivors was 20 days (IQR 15–27). Among this group, 15 (10.8%) experienced a relapse after a median of 169 days (IQR 146–281) from discharge. No significant differences were observed between HIV-positive and HIV-negative subgroups in terms of mortality, relapse rate, or time to relapse. Univariable and multivariable logistic regression models identified no significant predictors of disease relapse (Table 5), although there was a trend towards statistical significance for the positive association between time to remission and relapse (*P* = 0.084).

**TABLE 5:** Univariable and multivariable logistic regression models for the outcome variable; relapse.

Variable	Odds ratio	*P*-value	95% CI
**Univariable analysis**			
Age	0.95	0.145	0.89–1.02
Sex (female)	0.70	0.535	0.22–2.19
HIV status (positive)	2.40	0.412	0.30–19.39
Treatment (FFP and PEX)	1.15	0.841	0.30–4.41
Time to remission	1.05	0.084	0.99–1.10
**Multivariable analysis**			
Age	0.95	0.140	0.88–1.02
Sex (female)	0.50	0.285	0.14–1.78
HIV status (positive)	2.07	0.521	0.22–19.23
Treatment (FFP and PEX)	0.96	0.961	0.22–4.24
Time to remission	1.05	0.102	0.99–1.11

FFP, fresh frozen plasma; PEX, plasma exchange; CI, Confidence Interval.

Severe TTP was the primary cause of death in both HIV-positive and HIV-negative patients. Additionally, seven (5.9%) HIV-positive patients succumbed to sepsis, whereas no sepsis-related deaths occurred in the HIV-negative subgroup (Table 2).

## Discussion

This was a single-centre retrospective study which evaluated the clinical features, laboratory findings and outcomes of 139 patients diagnosed with idiopathic and HIV-associated TTP over a 10-year period during the ART era. The last study on TTP from our centre in Cape Town was conducted in 2005, before ART became widely available.^20^

Of the patients included in the study, the majority (85.6%) were HIV-positive, consistent with previous reports on TTP in South Africa.^13,20^ As of 2017, South Africa had the largest HIV burden globally, with an estimated 8 million South Africans living with HIV, which accounts for the high proportion of HIV-associated TTP observed in our setting.^23^ In contrast, idiopathic TTP is rare, both in South Africa and worldwide, with an annual incidence of approximately one case per million population.^6^ HIV infection increases the risk of developing TTP by up to 40-fold.^24^ Despite a 47% decline in new annual HIV infections between 2000 and 2019, the number of patients presenting annually with HIV-associated TTP at our centre remained unchanged (Online Appendix 1 Figure 1-A1).

The median age of diagnosis for patients with HIV-associated TTP was 34 years, similar to the findings in another centre in South Africa who reported a mean age of 33.7 years.^13^ This aligns with national data from Statistics South Africa, showing that HIV predominantly affects young adults aged 20–34 years.^23,17^ Among patients with HIV-associated TTP in our cohort, 73.4% were women, consistent with previous reports.^13,21^

In the HIV-associated TTP group, half were ART-naïve (representing first diagnosis and presentation of HIV), while the other half had defaulted ART at the time of presentation. The proportion of study patients who had defaulted ART varied from 18% to 79% annually, with no specific trend over time, but the proportion of patients who presented after defaulting ART was substantially higher than the 21.9% reported by Masoet, Bassa, and Chothia in 2019 and other centres across South Africa.^13^ Given that South Africa has the largest ART programme globally, it is notable that half of our HIV-infected patients presented after defaulting ART. These findings suggest potential gaps in counselling both at ART initiation and during follow-up in primary healthcare settings.^13,17^

The large proportion of ART-naïve patients at presentation can partly be explained by the historical evolution of the ART programme in the country. Prior to 2010, ART was initiated in patients with an absolute CD4+ T-cell count below 200 cells/µL or WHO-defined stage IV HIV (AIDS-defining illness). By 2013, the threshold CD4+ T-cell count was raised to 350 cells/µL, and in 2018, universal test-and-treat was implemented, with ART initiated irrespective of CD4+ T-cell count. Following this improvement to the ART programme, the number of patients presenting with AIDS-defining illnesses has declined.^17,23^ The median absolute CD4+ T-cell count for the HIV-positive cohort was 154.5 cells/µL, suggesting that HIV-associated TTP remains largely a disease of untreated HIV. Notably, only one patient in our study was HIV-positive with a suppressed viral load at presentation. In this case, TTP was attributed to newly diagnosed systemic lupus erythematosus.

Neurological dysfunction occurred more frequently in the HIV-positive subgroup (89.9%) than in the HIV-negative group (79%), although this difference was not statistically significant. Masoet et al.^13^ reported neurological dysfunction in 78% of their cohort, with no significant differences by HIV status. The reason for the higher proportion of neurological dysfunction in HIV-associated TTP in our study is unclear. Neurological dysfunction was significantly associated with failure to achieve remission, occurring in 100% of non-remitters versus 84% of those who achieved remission. This is consistent with the findings of Swart, Schapkaitz, and Mahlangu, who reported that impaired consciousness predicts poor outcomes.^19^ Our study did not differentiate between types of neurological dysfunction, which may influence prognosis, as mild delirium would likely resolve faster than severe deficits such as stroke or ongoing seizures.

Similar proportions of HIV-positive (72.3%) and HIV-negative (73.7%) patients achieved remission. Time to remission was also comparable between the two groups. In the HIV-positive cohort, the majority (83%) received FFP infusion only, while the remainder required both FFP infusion and PEX. These observations align with the study by Masoet et al. demonstrating similar remission rates regardless of HIV status or treatment modality. ART initiation was the only factor independently associated with remission in that study.^13^

Patients treated with FFPs only achieved remission after a median of 8 days from admission, whereas those who required both FFPs and PEX had a median recovery time of 12 days. This difference is not surprising, as PEX is typically reserved for patients with resistant disease. In comparison, Masoet et al. reported shortened times to remission (2 days in the FFP-only group and 4.5 days in the PEX and FFP group).^13^ These differences likely reflect variations in treatment protocols. In the Masoet study, PEX was available only every second day, whereas at our centre, PEX is given daily except on weekends, when large-volume FFPs are administered. Additionally, our centre uses larger plasma volumes of FFPs (30 mL/kg/day) compared to 20 mL/kg/day in the study by Swart et al.^19,21^; however, no South African study has directly compared these different dosing regimens. Such a study would be valuable to optimise PI dosing and prevent over-treatment and under-treatment.

During the study period, 45 patients (37.8%) with HIV-associated TTP demised, and two patients (1.7%) were presumed dead based on laboratory results from subsequent admission at another healthcare facility. These findings are comparable to the mortality rate of 43.9% reported by Masoet et al. In that study, all patients received PI only, unless the patients had refractory disease, in which case PEX was administered on alternate days.^13^ The majority (81.5%) of deaths during the first admission were because of severe disease, which is consistent with the high mortality of TTP, reported to remain around 20%, even with adequate treatment.^15,25^ Differences in treatment protocols across South Africa make comparisons of treatment outcomes between centres challenging.

The main limitations of this study relate to its retrospective design and limited diagnostic confirmation. ADAMTS13 activity and inhibitor testing were not routinely available during the study period, with fewer than 5% of patients tested, precluding assessment of their diagnostic and prognostic significance and raising the possibility of both under-diagnosis and over-diagnosis of TTP. This is particularly relevant in HIV-associated disease, where endothelial injury from opportunistic infections such as cytomegalovirus can result in a TTP-like syndrome. The retrospective nature of the data also limited precise assessment of disease severity, including neurological and renal dysfunction. Pregnant patients with thrombocytopenia and fragmentation haemolysis resulting from pregnancy-related hypertensive disorders were investigated and followed up for clinical resolution to exclude TTP. However, some of these patients received high doses of FFPs during the first few days of admission, which may have obscured TTP-related disease. Conversely, TTP may have been over-diagnosed, particularly in patients with features suggestive of a TMA who were not reviewed by Clinical Haematology and also had no ADAMTS13 testing performed.^4^ Finally, temporal trends in HIV-associated TTP before and after implementation of the national test-and-treat strategy could not be evaluated, as dates of HIV diagnosis and ART initiation were inconsistently recorded; prospective studies are needed to address this.

## Conclusion

This study represents the largest reported cohort of HIV-associated TTP patients in South Africa during the ART era. Despite advances in ART and supportive care, TTP remains associated with high mortality, irrespective of HIV status. Limited access to plasma therapy, which is available only at tertiary centres in South Africa, delays treatment initiation, highlighting the importance of rapid diagnosis and early FFP administration. ART adherence is critical, as treatment interruptions contribute to TTP relapse. Strengthening early HIV management, improving ART adherence and expanding access to plasma therapy may help reduce mortality and shorten hospital stays for patients with TTP.
